# Numerical and Experimental Investigations on Residual Stress and Hardness within a Cold Forward Extruded Preform

**DOI:** 10.3390/ma16062448

**Published:** 2023-03-19

**Authors:** Tae-Wan Ku

**Affiliations:** Engineering Research Center of Innovative Technology on Advanced Forming, Pusan National University, Geumjeong-gu, Busan 46241, Republic of Korea; longtw@pusan.ac.kr; Tel.: +82-51-510-3130

**Keywords:** residual stress, plastic deformation damage, hardness, microstructure, finite element simulation, cold forward extrusion

## Abstract

Using a preform fabricated by a cold forward extrusion process, the present study numerically predicted and experimentally investigated its residual stress and microstructural characteristics, as well as its plastic deformation damage and hardness. Prior to realizing the preform, AISI 1035 cold-drawn medium carbon steel material with a diameter of 50.0 mm and a height of 121.0 mm is first spheroidized and annealed, after which phosphophyllite is used to coat its outer surface. To identify the influence of the spheroidizing and annealing on the mechanical properties and the microstructural phase, uniaxial compression tests and microscopic observations are carried out. After assuming the deformation behavior of the workpiece during the cold forward extrusion with a plastic material model and with an elasto-plastic material model, separately, three-dimensional finite element simulations are adopted to visualize the residual stress and the plastic deformation damage. The preform produced by cold forward extrusion is fully scanned by using an optical 3D scanner, the Vickers micro-hardness is measured, and the residual stress through EBSD (electron backscatter diffraction) analysis is observed. Briefly, the results show that the ferrite and pearlite within the raw workpiece is well spheroidized by the heat treatment, and that there is a decrease in the KAM (kernel average misorientation) value of about 40%. In terms of the preform obtained by the cold forward extrusion, the dimensional requirement is more suitably met with the predicted layout when adopting the elasto-plastic material model than that of the plastic material one, and the numerically predicted residual stress agrees with the Vickers micro-hardness distribution. It can be verified that the dislocation density (or the internally stored strain energy) based on the IQ map and the IPF map is substantially increased around the extrusion region, and that the KAM value is increased by roughly 516% as the whole average of the observed values.

## 1. Introduction

To enable a fast response to industrial demands such as minimizing lead times and production costs, which is necessary for tool development and prototype fabrication in a cold forging operations, and for product quality verification, various digital twin models have come to be used to assess a plastic’s deformation behavior and its stepwise history of workpiece materials by making virtual predictions and visualizations through a series of FEM (finite element method)-based numerical simulations in implementations that are as similar as possible to an actual forging process [1,2,3,4]. From the perspective of plastic deformation in the die cavity, proceeding continuously from an initial billet to a target shape, and of the post deformation occurring when a cold deformed product is pulled out from the cavity, and based on empirical assumption that elastic recovery of the workpiece material is small enough to be ignored, numerical predictions and quick visualizations using a series of plastic (or rigid-plastic) material models have been widely and commonly adopted to satisfy the aforementioned industrial needs [5,6,7,8,9]. 

There have been increasing interests in the durability and stiffness of metal forged members. Recently, in the conceptual process design step involved in the intermediate shape and practical manufacturing process, there have been many efforts to guarantee product quality that have been devoted to diverse considerations of minimizing the residual stress level and the plastic deformation damage degree, as well efforts to suppress these local concentrations [10,11,12]. However, it is well known that the numerical approaches depend upon the plastic material models, which are limited in their ability to predict and evaluate the residual stress within the cold forged product, due to the fact that the elastic deformation behavior is excluded in view of the constitutive equation, which is often called a flow stress model [13,14,15,16,17,18,19]. Consequently, a series of predictions and evaluations of the residual stress level have been attempted by adopting elasto-plastic material models [20,21,22]. A preform is widely used in a one-shot cold forging process to manufacture a three-dimensionally complicated target shape, and this is considered to cause high-leveled residual stress and critical forging defects [23,24]. 

The preform dealt with in this study is also an intermediate workpiece fabricated through a cold forward extrusion process in order to produce a drive shaft as one of internal metal members that constitute an industrial hydraulic pump. Prior numerical and experimental studies related to the two-stage cold forging process for developing the drive shaft did not deal with the residual stress and the plastic deformation damage, including the hardness variation, because they were only focused on the stress distribution and the plastic deformation behavior, as well as the dimensional assessment and the forging load prediction [25,26,27]. However, when considering the operating environment of the drive shaft, it has to satisfy a basic series of critical requirements such as sufficient structural stiffness and mechanical strength against torque, substantial durability, and so forth. To this end, the residual stress and the plastic deformation damage within the preform as the intermediate workpiece of the drive shaft have to be managed at an appropriate level [28,29,30,31,32]. 

This study investigated the residual stress and the microstructural characteristics, as well as the plastic deformation damage and the hardness, within the preform manufactured through the cold forward extrusion operation. First, the raw workpiece of AISI 1035 cold-drawn medium carbon steel with a diameter of 50.0 mm and a height of 121.0 mm was heat treated through spheroidizing and annealing to improve its forgeability and ductility. In order to identify the influences of the heat treatment on the mechanical properties and the material characteristics, the microstructural phase and the grain size were observed, and uniaxial compression tests were also carried out. Using digital twin models in which the cold forward extrusion process was segmented into the forward extrusion and the ejection, and where the deformation behavior of the heat-treated workpiece was respectively assumed using the plastic material model and the elasto-plastic material one, FEM-based numerical simulations were conducted to predict the residual stress and the plastic deformation damage within the workpiece material. Moreover, the preform was manufactured in the cold forward extrusion experiment, and fully captured using an optical 3D scanner, after which the dimensional soundness was compared between the required specifications and the numerically simulated results. The residual stress and the plastic deformation damage predicted by the numerical simulation were also evaluated and compared with those observed by using EBSD (electron backscatter diffraction) analysis and Vickers micro-hardness test equipment. Resultantly, it was confirmed that the residual stress predicted through the cold forward extrusion simulation was distributed similarly to the micro-hardness distribution observed by the Vickers micro-hardness measurement, and that its level appeared to be relatively alike when compared with the KAM value obtained by the EBSD analysis. 

## 2. Microstructural Characteristics and Mechanical Properties

### 2.1. Preform Forging Conditions

**Figure 1:**
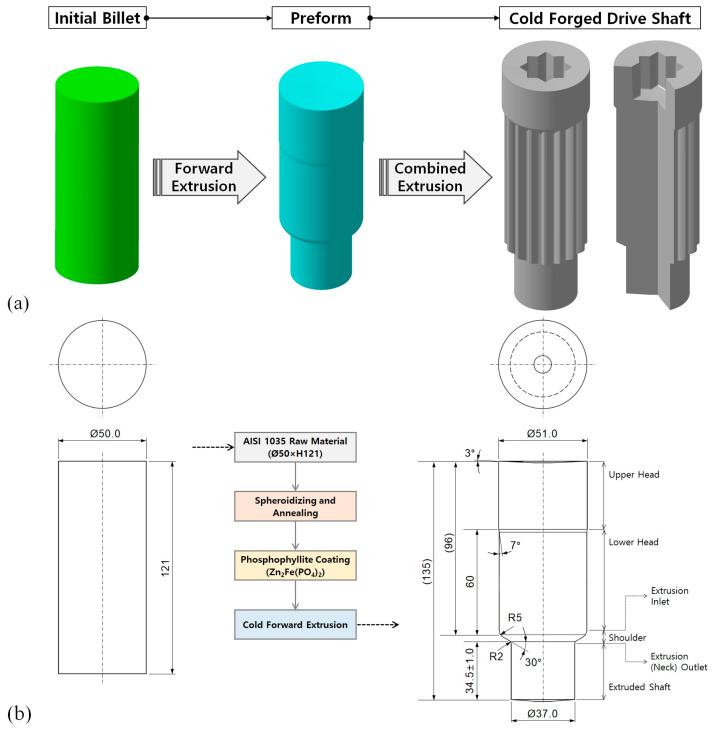
Schematic illustrations of (**a**) the overall process flow for manufacturing a drive shaft, and (**b**) a detailed flowchart for fabricating preform (unit: mm).

Figure 1a presents a sequential flow of the two-stage cold forging process used to produce the drive shaft, in which an internal spline with an irregular hexadecagonal cross-section and a sixteen-tooth spur gear were merged into a single metal member along a neutral axis. It can be seen that the round-type initial billet is shaped to the preform through the cold forward extrusion, and the preform is again deformed through the cold combined extrusion, which is defined as a forward–backward forging process, to obtain the cold forged drive shaft [25,26,27]. Given that the preform fabrication is the main object of this study, the subspecialized process flow is illustrated in Figure 1b. The cold-drawn round bar of the AISI 1035 medium carbon steel, the chemical composition of which is summarized in Table 1, was selected as the workpiece material due to the facts that it has many successful applications to mechanical-structural metal members in various industrial fields. However, because of irregular and uneven microstructural grain shapes within the cold-drawn raw material, and due to inherent residual stress, a series of critical cold forging failures such as micro-crack and plastic deformation defects can result from the raw material being used directly in the cold forging operation.

**Figure 2:**
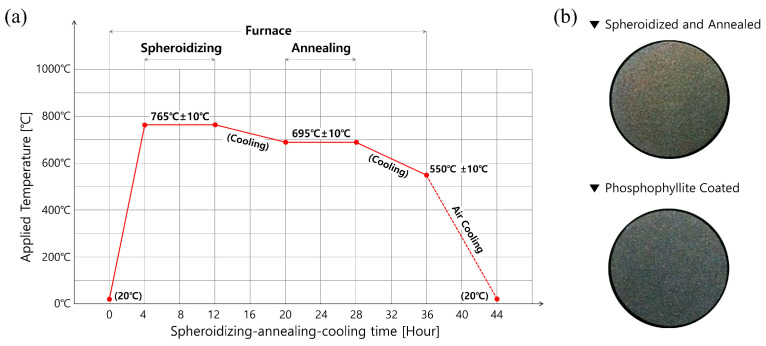
(**a**) Time–temperature history of spheroidizing and annealing, and (**b**) heat-treated and phosphophyllite-coated AISI 1035 medium carbon steel workpiece.

In order to resolve these issues, heat treatments for the raw workpiece material were considered, as shown in Figure 2a. That is, to more evenly modify and regularly rearrange the microstructures, spheroidizing heat treatment for 8 h at 760 °C ± 10 °C was first applied to the workpiece, which had an initial diameter of 50.0 mm and a height of 121.0 mm [33,34]. Then, to improve the mechanical properties and the ductility such as the yield stress and the ultimate strength as well as the elongation, annealing heat treatment for 8 h at 695 °C ± 10 °C was also conducted in the same furnace. Then, the material was taken out into the atmosphere when the workpiece temperature reached about 550 °C ± 10 °C through furnace cooling, and it was further cooled down to about 20 °C through air cooling for 8 h. As another consideration, for the purpose of improving the frictional behavior on the contact interfaces between the workpiece and the tool surfaces during the cold forward extrusion, phosphophyllite (Zn_2_Fe(PO_4_)_2_) was applied coating for 1 h at about 80 °C. This was done to form a coating layer with a thickness of roughly 1.0 µm~3.0 µm on the previously heat-treated workpiece, and thus a shear frictional coefficient of 0.098 can be expected. Figure 2b shows the spheroidized-annealed workpiece and the phosphophyllite-coated initial billet.

### 2.2. Microstructural Characteristics

**Figure 3:**
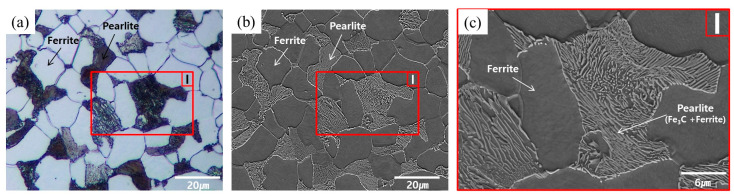
Microstructures of raw material: (**a**) OM image (×1000); (**b**) SEM image (×1000); (**c**) SEM image (×3000).

**Figure 4:**
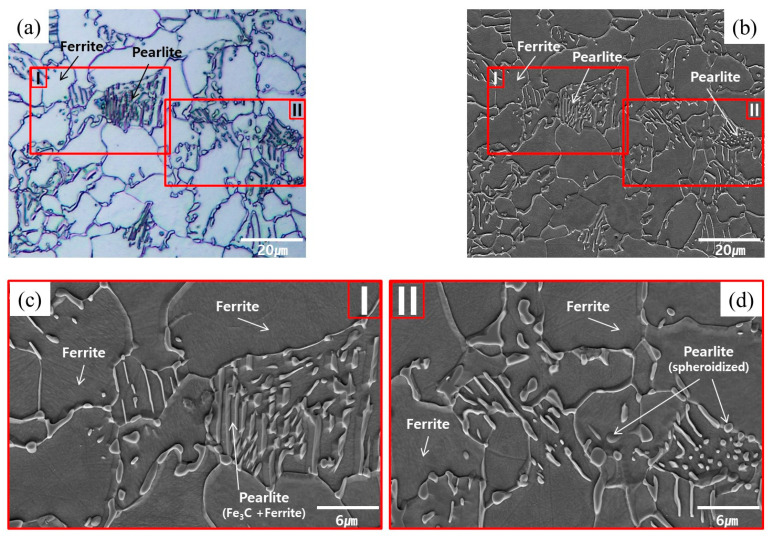
Microstructures of spheroidized and annealed specimen: (**a**) OM image (×1000); (**b**) SEM image (×1000); (**c**) SEM image in section I (×3000); (**d**) SEM image in section II (×3000).

The workpiece specimens before and after the spheroidizing and annealing were etched with 3% Nital solution to allow for the grain shape and size to be measured and compared, and to verify the microstructural phase. For the AISI 1035 raw material before any heat treatment, Figure 3a,b illustrates the microstructures captured with a high magnification factor of 1000 using an Olympus BX51M OM (optical microscopy) apparatus and Carl Zeiss Supra 45 SEM (scanning electron microscopy) equipment, and Figure 3c depicts the SEM microscopic photograph magnified by the factor of 3000, on the captured part as I. In perspective of the spheroidized and annealed specimen, Figure 4a,b presents the microscopic images with the same magnification factor of 1000, while Figure 4c,d shows the enlarged SEM images on the denoted sections as II and III. Based on the standard test methods used to determine average grain size (ASTM E112-13), the microstructures of the raw material were found to be densely distributed with an average value of about 8.84 µm, as shown in Figure 3a. For the spheroidized and annealed specimen, the microstructures were also observed to be well distributed, but the average grain size due to grain coarsening was slightly increased with an average value of approximately 9.36 µm, as shown in Figure 4a. The grain sizes within the raw and the spheroidized-annealed specimens were summarized in Table 2. Regarding the microstructural phase in the raw specimen, Figure 3 clearly shows a two-phase microstructure composed of the polygonal ferrite and the pearlite with the lamellar structure of the ferrite and the cementite (Fe_3_C). For the spheroidized and annealed specimen, Figure 4 depicts the two-phase microstructure, but it can also be seen that the pearlite with the lamellar structure in Figure 3b,c was decomposed. For further scrutiny, Figure 4c,d shows that the polygonal ferrite is freely distributed and that the decomposed pearlite is partly broken and partially spheroidized, which means that the flake-phase cementite (Fe_3_C) is well spheroidized.

### 2.3. Mechanical Properties

In most forging processes, including the cold forward extrusion dealt with in this study, it is well known that the workpiece materials suffer from severe compressive deformation. Accordingly, it was necessary to verify the compressive strengths and build up a criterion with which to analyze the validity of the stress level as predicted from a series of numerical simulations, so uniaxial compression tests were conducted on the AISI 1035 raw and the heat-treated workpiece materials using the circular-type specimens with a diameter of 8.00 mm and a height of 6.40 mm, these were prepared by referring to the standard test methods of ASTM E9-09. A universal material testing apparatus of INSTRON 5988 with a force capacity of nearly 400 kN was also used under the experimental conditions of a uniform compressing speed of 6.00 mm/min at room temperature.

**Figure 5:**
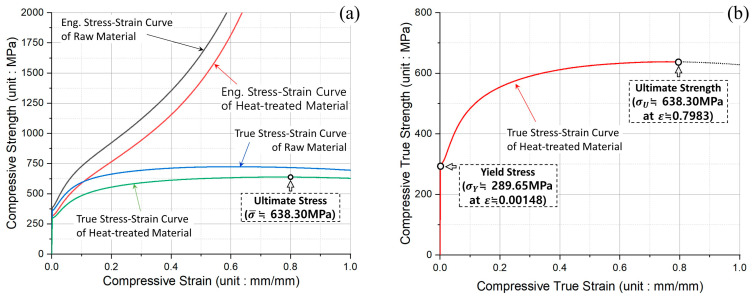
Stress-strain curves extracted by uniaxial compression tests: (**a**) compressive stress-strain curves of raw and spheroidized-annealed specimens; (**b**) compressive true stress-strain curve of spheroidized and annealed specimen.

The compressive force and the displacement data that were measured through the uniaxial compression tests were first converted to the engineering stress-strain data, and then the engineering stress-strain data were again transformed to the true stress-strain data. As the experimental results in Figure 5a illustrate, the compressive stress-strain curves for the raw and the heat-treated specimens, and it was demonstrated that the raw material has sufficient compressive strength, and that the heat-treated workpiece has reasonably softened to the point that it can be used as the initial billet material. Further, the mechanical properties obtained from the uniaxial compression tests are summarized in Table 3. Regarding the true stress-strain data extracted from the uniaxial compression experiments, the compressive yield stresses for the raw material and the spheroidized-annealed specimen were gauged at roughly 342.53 MPa and about 289.65 MPa, respectively, and the ultimate strengths were also taken to be approximately 723.99 MPa and nearly 638.30 MPa, respectively. For the results induced by applying the spheroidizing and annealing heat treatment, the yield stress and the ultimate strength were found to be decreased by about 50 MPa and roughly 85 MPa, compared to the values of the raw material. The compressive true stress-strain curve was finally obtained, as presented in Figure 5b. With respect to the cold forging process, the large elongation property is basically required to ensure the forgeability and the workability. However, because the elongation of 78.93% within the compressive true stress-strain curve demonstrated in Figure 5b was regarded to be slightly lacking for achieving good convergence and iteration during three-dimensional FE simulations, the flow stress model was contemplated to assess whether it could realize the work-hardening behavior, where the true stress gradually increased after the ultimate strength point. 

**Figure 6:**
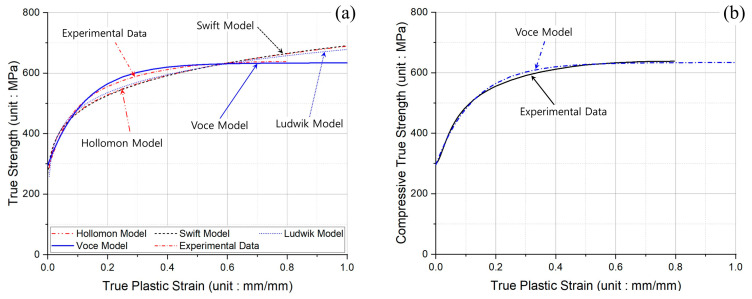
Comparison of flow stress curves using experimental data from uniaxial compression tests: (**a**) true plastic stress-strain curves using various flow stress models; (**b**) true plastic stress-strain curves between Voce model and experimental data.

Because the uniaxial compression tests on the spheroidized and annealed specimens were performed at the conditions of room temperature (20 °C) and a constant test speed (6.00 mm/min), the four representative flow stress models were considered with reference to the true stress-strain curve presented in Figure 5b [13,14,15,16,17,18,19]. Consequently, Figure 6a depicts the flow stress curves plotted by adopting the four constitutive equations, and it was then shown that the flow stress curve predicted by Voce’s model was more consistent than the others. The optimized equations with regard to the flow stress models are also summarized in Table 4, and the true plastic stress-strain curves derived by Voce’s formula and the uniaxial compression tests are presented in Figure 6b. As a result, the flow stress relation as Voce’s exponential formula was applied to FEM-based numerical simulations related to the cold forward extrusion process in this study.

## 3. Numerical Simulations of Cold Forward Extrusion

### 3.1. Numerical Simulation Conditions

**Figure 7:**
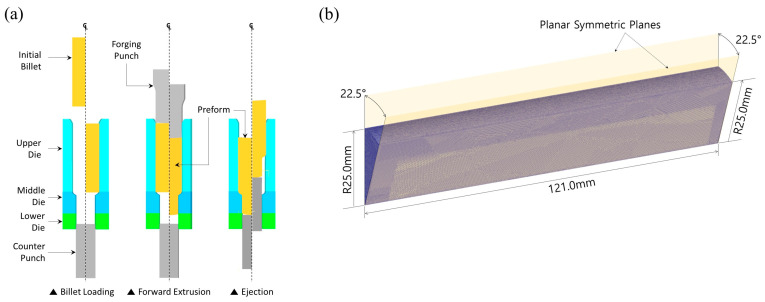
(**a**) FE simulation flow of cold forward extrusion for shaping preform, and (**b**) discretized mesh structure of initial billet with one-sixteenth planar symmetric condition.

The most efficient method for shortening the computation time of the numerical simulation is to use a minimal symmetric structure wherein the deformation behavior can equally be implemented in comparison to that of the whole geometry. As presented in Figure 1b, it was indicated that the preform can be regarded as the axisymmetric condition, so two-dimensional FE (finite element) modeling is available. However, considering the disparate features of the internal spline and the spur gear geometries that are ultimately required within the cold forged drive shaft shown in Figure 1a, it was revealed that the internal spline geometry with the irregular hexadecagonal cross-section has a one-sixteenth (1/16) planar symmetric structure. It was also revealed that the spur gear with the sixteen-tooth profile has a one-thirty-second (1/32) planar cyclic symmetric configuration, so the minimized symmetric condition is regarded as a one-sixteenth (1/16) planar symmetric one. In other words, considering that the two-stage cold forging process for producing the drive shaft is carried out continuously, it is also desirable to keep the FE models for the numerical simulations to maintain the same symmetric condition. Consequently, a one-sixteenth planar symmetric FE model for the cold forward extrusion was adopted for the forging punch and the extrusion die, as well as for the initial billet [25,26,27,28]. Figure 7a depicts the three-dimensional FE models and the numerical simulation steps of the cold forward extrusion in order to visualize the preform. For further particulars, the initial billet to which the phosphophyllite coating was applied after the spheroidizing and annealing heat treatment is fed into the die cavity, and the cold forward extrusion operation is performed, after which the cold deformed preform is taken out by operating the counter punch. Here, due to the fact that the feeding step only means carrying the initial billet into the die cavity without any deformation, the forward extrusion sequence and the ejection operation were regarded as the subjects of interest for a series of the numerical simulations.

The mesh structure of the initial billet in the FE models with one-sixteenth (1/16) planar cyclic symmetry was discretized using 648,489 first-order tetrahedral elements as illustrated in Figure 7b, and an automatic remeshing technique was adopted. The automatic remeshing option supported by DEFORM-3D^TM^ (Scientific Forming Technology Corporation, Columbus, OH, USA) was adopted for visualizing the cold forward extrusion of the preform in this study because it locally reconstructs the mesh structure around the elements where severe distortion as well as warpage and folding occur, and the whole mesh structure and the number of the discretized meshes do not change dramatically. As for other simulation conditions, the effect of the strain rate was neglected, because the numerical simulation related to the cold forward extrusion was defined as a quasi-static process. When the phosphophyllite (Zn_2_Fe(PO_4_)_2_) coating was applied on the outer surfaces of the spheroidized and annealed workpiece material, it was expected that the coating layer with a thickness of about 1.0 µm~3.0 µm was formed, and then the friction behaviour was experimentally known to be 0.098. Therefore, the friction parameter in the contact interface between the tool surfaces and the billet material was defined by 0.098 as the shear friction coefficient.

In addition to the elastic mechanical properties summarized in Table 3, the flow stress relation that was built up based on the uniaxial compression tests was applied to the three-dimensional FEM-based numerical simulations. In order to realize the cold forward-extruded preform as well as to predict and compare the residual stress, the deformation behavior of the workpiece material during the three-dimensional FEM-oriented numerical simulations were specially classified using the plastic and the elasto-plastic material models. On the other hand, the Cockcroft and Latham’s ductile damage criterion was selected to predict and determine the plastic deformation damage degree within the workpiece material during the cold forward extrusion operation [35,36,37].

### 3.2. Numerical Simulation with Plastic Material Model

**Figure 8:**
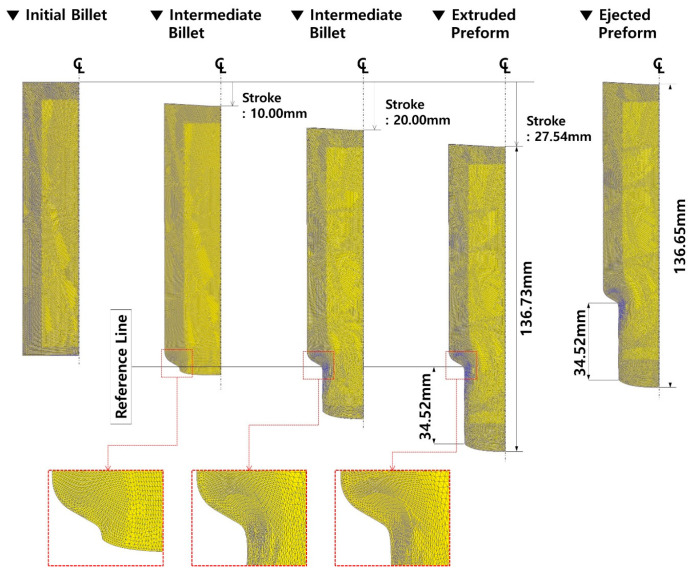
Deformation history during forward extrusion and ejection in the case where plastic material model was applied.

After assuming the deformation behavior of the initial billet using the plastic material model, the three-dimensional numerical simulations related to the forward extrusion process and the ejection operation were progressively performed, and the predicted results on the effective stress and the residual stress, as well as the plastic deformation damage, are presented in Figure 8. Specifically, it shows the deformation history of the intermediate workpiece including the mesh structure and its distribution, when the punch stroke reached 10.00 mm, 20.00 mm, and 27.54 mm. Here, the total stroke of the forging punch to satisfy the extrusion length of 34.5 mm ± 1.00 mm was required to be nearly 27.54 mm. It was also revealed that the meshes around the extrusion outlet (neck) region were severely distorted and densified, and that the plastic deformation was concentrated in this area. When the extruded preform was pulled out from the die cavity by activating the counter punch, it was noted that the mesh structure was without any critical change. In view of the longitudinal length measured along the central line, the whole length of 136.73 mm was slightly shortened to be 136.65 mm, with a variation of 0.08 mm as a result of the counter punch being operated to eject the deformed preform from the die cavity. 

**Figure 9:**
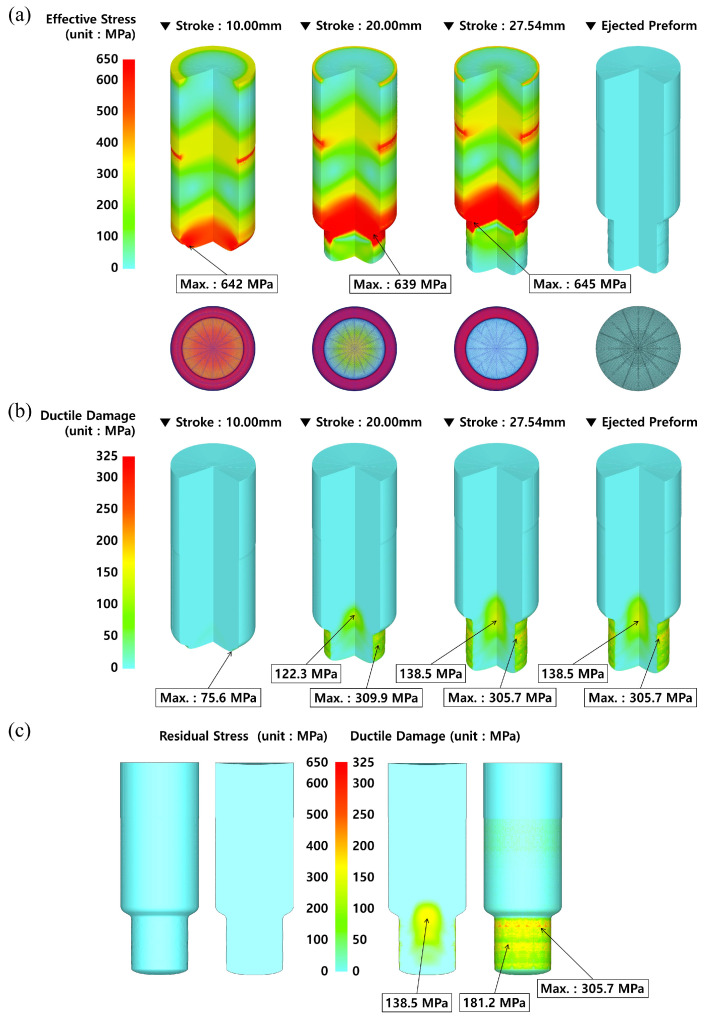
Numerical simulation results in the case where plastic material model was applied: (**a**) effective stress distribution; (**b**) plastic deformation damage distribution; (**c**) distribution of residual stress and plastic deformation damage after preform ejection.

Figure 9a indicates the distributions of the effective stress, and it was predicted that the maximum effective stresses for the punch strokes of 10.00 mm, 20.00 mm, and 27.54 mm were roughly 642 MPa, 639 MPa, and 645 MPa, respectively, around the neck region of the billet being extruded. It was pointed out that the maximum equivalent stresses occurred around the extrusion outlet (neck) area, because the compressive deformation was locally concentrated in accordance with the fact that the cross-sectional area was rapidly decreased with the extrusion ratio of about 1.87 (i.e., the reduction ratio of the cross-sectional area on the extrusion inlet and the outlet). Further, due to the fact that the FE simulation of adopting the plastic (or the rigid-plastic) material model cannot provide the residual stress when the applied external load is released, the effective stress as the residual stress inherent in the preform that was fully ejected from the die cavity was not observed. On the other hand, the plastic deformation damage was also evaluated using the Cockcroft and Latham’s ductile damage criterion, and its distribution was depicted in Figure 9b. When the punch strokes reached 10.00 mm, 20.00 mm, and 27.54 mm, the maximum plastic deformation damage values were distributed at nearly 75.6 MPa, 309.9, and 305.7 MPa, respectively, on the surface section of the extruded shaft. These higher values were attributed to the excessive plastic deformation and the frictional influence on the contact interface between the workpiece and the middle (extrusion) die. The plastic deformation value was also evaluated as approximately 305.7 MPa on the surface region of the extruded shaft after the ejection process was fully completed. In addition, it was shown that a plastic deformation damage of nearly 138.5 MPa was distributed at the inner central part around the extrusion neck region. In the cold forward-extruded preform, because the damage level in the inner central region was more important than that on the surface of the extruded shaft, it was necessary to take note of the damage level in the inner central section.

In further detail, Figure 9c depicts the distribution of the residual stress and the plastic deformation damage when the ejection process was fully completed. Here, the residual stress did not appear due to the aforementioned disadvantage. However, the plastic deformation damage predicted by using the Cockcroft and Latham’s ductile damage criterion was found to be highly distributed at nearly 306.8 MPa on the external surface of the extruded shaft, and to be roughly 138.5 MPa at the inner central part around the extrusion neck region. These high-level plastic deformation damage values were regarded to be caused by following reasons: (1) the ductile damage value of about 305.7 MPa on the external surface was induced by the severe deformation of the meshes, due to the fact that the compressive load and the friction resistance were increased in accordance with the decrease in the cross-sectional area at the extrusion outlet, and (2) a damage value of nearly 138.5 MPa in the central part was derived by the concentration of the compressive force as the influence from the reduction of the cross-sectional area [31].

### 3.3. Numerical Simulation with Elasto-Plastic Material Model

**Figure 10:**
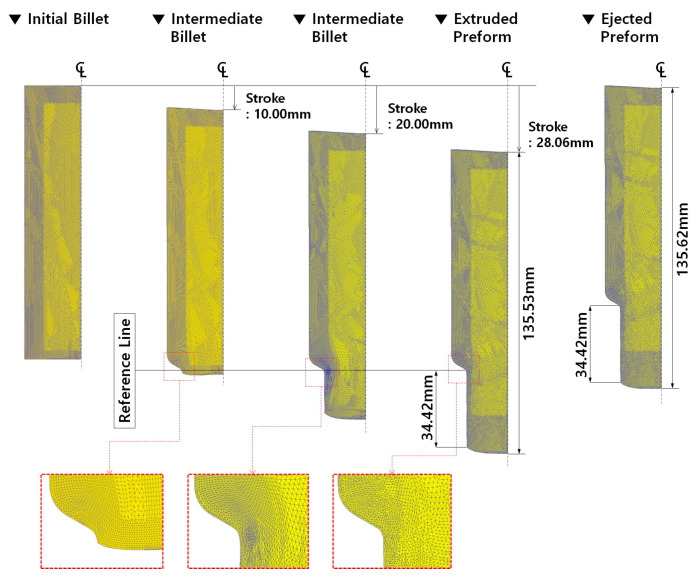
Deformation history during forward extrusion and ejection in the case where elasto-plastic material model was applied.

**Figure 11:**
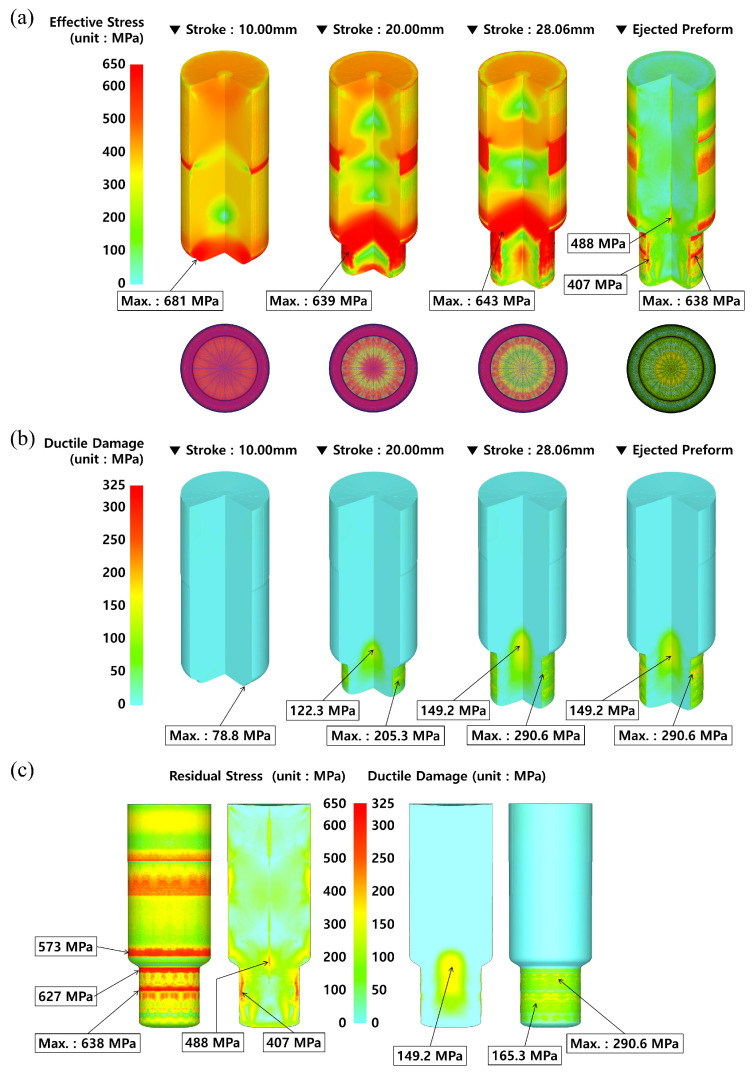
Numerical simulation results in the case where elasto-plastic material model was applied: (**a**) effective stress distribution; (**b**) plastic deformation damage distribution; (**c**) distribution of residual stress and plastic deformation damage after preform ejection.

The material model was redefined as the elasto-plastic material model with the same simulation conditions as when applying the plastic material model, and the cold forward extrusion simulations were reperformed. Figure 10 illustrates the simulated results such as the effective stress and the residual stress distributions, along with the plastic deformation damage. Before looking into the effective stress and the residual stress distributions, the deformed configurations and the rediscretized mesh structures at the punch strokes of 10.00 mm, 20.00 mm, and 28.06 mm are provided in Figure 11a. Here, the total stroke required to meet the extrusion length of 34.5 mm ± 1.00 mm was 28.06 mm, and then the numerically extruded shaft length was about 34.42 mm. It was shown that the meshes around the extrusion outlet (neck) were severely densified and distorted, and the plastic deformation was accumulated in this section. On the whole, the deformation history and the mesh structures when the elasto-plastic material model was adopted into the three-dimensional cold forward extrusion simulation were similar compared to those when the plastic material model was applied. With respect to the longitudinal length measured along the central line, the whole length of 135.53 mm was slightly enlarged to be 135.62 mm with a difference of 0.09 mm created by the elastic recovery while the counter punch was enacted in order to pull the deformed preform out from the die cavity.

As shown in Figure 11a, the maximum effective stress was detected to be slightly large at about 681 MPa around the extrusion neck region with a punch stroke of 10.00 mm. When the stroke was 20.0 mm, it was predicted to be nearly 639 MPa on the surface of the extruded shaft, and in the case that the cold forward extrusion was finished with a stroke of 28.06 mm, the maximum equivalent stress was distributed to be roughly 643 MPa around the extrusion neck area. On the other hand, when the preform was fully ejected from the die cavity, the maximum effective stress was calculated to be about 638 MPa on the surface of the extruded shaft, but the stress was roughly 407 MPa in the inner area of the shaft, and nearly 488 MPa in the inner central section around the extrusion neck. Due to the fact that the residual stresses still remained in the preform even after the ejection operation was fully completed, the equivalent stress induced by the cold forward extrusion simulation using the elasto-plastic material model can be regarded as the residual stress. Accordingly, it was noted that the residual stress can be predicted through the numerical simulation using the elasto-plastic material model.

Figure 11b demonstrates the plastic deformation damage distribution induced from the Cockcroft and Latham’s ductile damage criterion, and the maximum values were mainly observed on the outer surface of the extruded shaft, and these were numerically observed as 78.8 MPa, 205.3 MPa, and 290.6 MPa for the punch strokes of 10.00 mm, 20.00 mm, and 28.06 mm, respectively. Moreover, these results revealed that the plastic deformation damages were distributed around the outer surface of the extruded shaft. When the deformed preform was fully ejected from the die cavity, the Cockcroft and Latham’s ductile damage was detected to be approximately 290.6 MPa as the maximum value, while it was predicted to be about 149.2 MPa in the inner central section around the extrusion neck. After the ejection step was completed, the predicted results of the residual stress and the plastic deformation damage are presented in Figure 11c. Then, the locally distributed residual stresses of roughly 627 MPa and 638 MPa at the upper and the middle surface of the extruded shaft, respectively, were visualized. It was particularly notable that the inherent stress of nearly 407 MPa was predicted within the inner side on the external surface of the extruded shaft, and that the residual stress of approximately 488 MPa was distributed at the inner core area of the preform shoulder between the extrusion inlet and the outlet. In view of the plastic deformation damage distribution, the damage degrees of about 290.6 MPa and 149.2 MPa appeared locally on the external surface and the inner core region of the extruded shaft, respectively, and these values were similar to those in the case of adopting the plastic material model. It can be also conjectured to be a result of similar reasons as with the results obtained in the case of applying the plastic material model in the forward extrusion and the ejection process. Consequently, the average residual stress level in the whole region of the preform ejected from the die cavity was dramatically decreased to under roughly 120 MPa, aside from that on the outer surface of the extruded preform. Remarkably, the residual stress can be numerically predicted by adopting the elasto-plastic material model, unlike in the case of the plastic material model [38].

### 3.4. Forging Load Prediction

**Figure 12:**
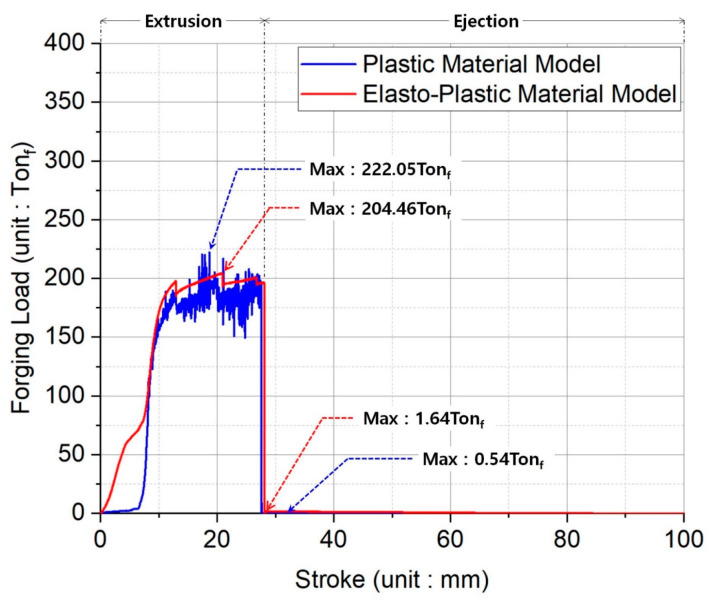
Forging load prediction required for forward extrusion and ejection.

In order to select a cold forging press with appropriate capacity, it is first necessary to predict the compressive load that can deform the initial workpiece to the required preform, and then consider a series of load dispersions to adjunct devices, and finally ensure a sufficient safety margin. When considering these requirements, the forging loads as the reaction forces induced by the forging punch for the forward extrusion and the counter punch for the ejection could be extracted. Figure 12 provides the forging loads required for the forward extrusion and the ejection processes. When the plastic material model was applied in the related simulations, the maximum loads were about 222.05 Ton_f_ for the forward extrusion and nearly 0.54 Ton_f_ for the ejection. 

However, when the elasto-plastic material model was adopted, the maximum loads were approximately 204.46 Ton_f_ for the forward extrusion and roughly 1.64 Ton_f_ for the ejection. During the forward extrusion, the peak load that was obtained through a calculation achieved by applying the elasto-plastic material model was found to be about 10% less than that achieved by adopting the plastic material model, but the maximum load required for the ejection was calculated to be roughly three times more. Consequently, for the forging press with the consideration of the structural safety margin, it is desirable to have over 500 Ton_f_ as the minimum capacity, and the ejection load by the counter punch must be at least 3 Ton_f_.

## 4. Results and Discussions

### 4.1. Preform Fabrication

**Figure 13:**
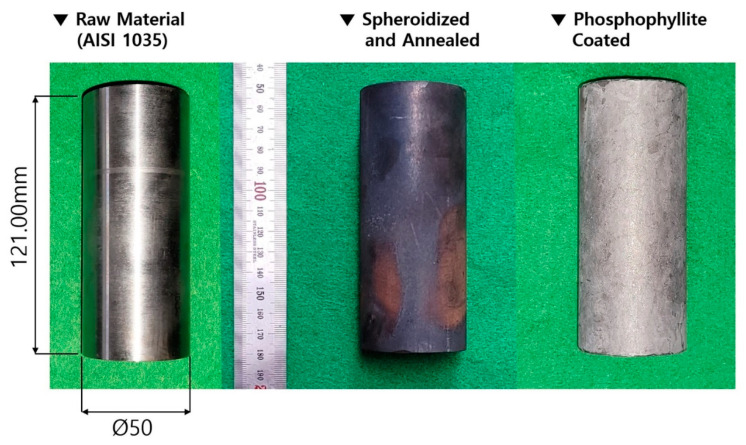
Initial workpiece, spheroidizing-annealing heat-treated specimen, and phosphophyllite-coated initial billet.

**Figure 14:**
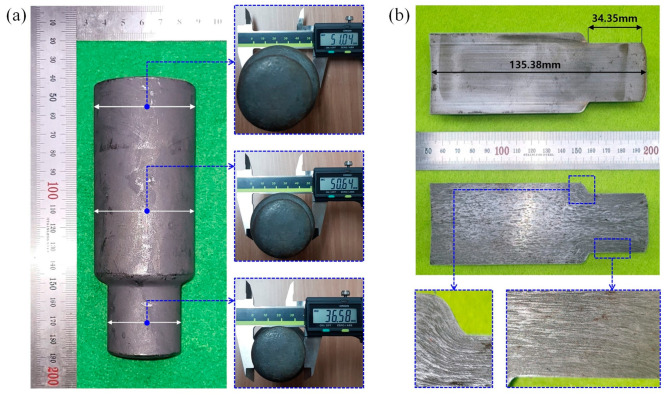
Fabricated preform and metal flow: (**a**) outer diameter of cold forward-extruded preform (unit: mm) and (**b**) metal flow on longitudinal cross-section.

Using the initial billet with a diameter of 50.0 mm and a height of 121.00 mm that was prepared with a phosphophyllite coating on the outer surface of a spheroidized and annealed specimen as presented in Figure 13, the cold forward extrusion experiment was carried out for fabricating the preform. Considering that a press capacity of over 500 Ton_f_ was predicted numerically for the cold forward extrusion, a hydraulic press with a capacity of 1300 Ton_f_ (OKP1250 of Sack and Kiesselbach) was used to produce the preform, and its counter punch ejecting load was about 5 Ton_f_. Figure 14a depicts the cold forward-extruded preform, and it appears that the preform was well forged in view of the whole feature, and that the phosphophyllite coating layer still remained on the outer surface. Further, the dimensions required for the preform as described in Figure 1b were also well matched with the measured outer diameters of 51.04 mm for the outer diameter at the upper head, 50.64 mm at the lower head, and 36.58 mm at the extruded shaft, as well as the gauged lengths of 135.38 mm along the center line and 34.35 mm along the extruded shaft. The whole feature and its longitudinally wire-cut cross-section of the cold forward-extruded preform, as well as the metal flow visualized by the corrosion test, are depicted in Figure 14b. In particular, the metal flow visualized through the corrosion test did not clearly appear because the plastic deformation was not large within the upper and the lower sections of the preform head. However, the flow lines at the preform shoulder part (that is, the extrusion region), where the plastic deformation from forward extrusion was mainly induced, were observed to be closely distributed, and these dense metal flow lines were still maintained at the inside part of the extruded shaft. 

### 4.2. Compatibility of Shape and Dimension

**Figure 15:**
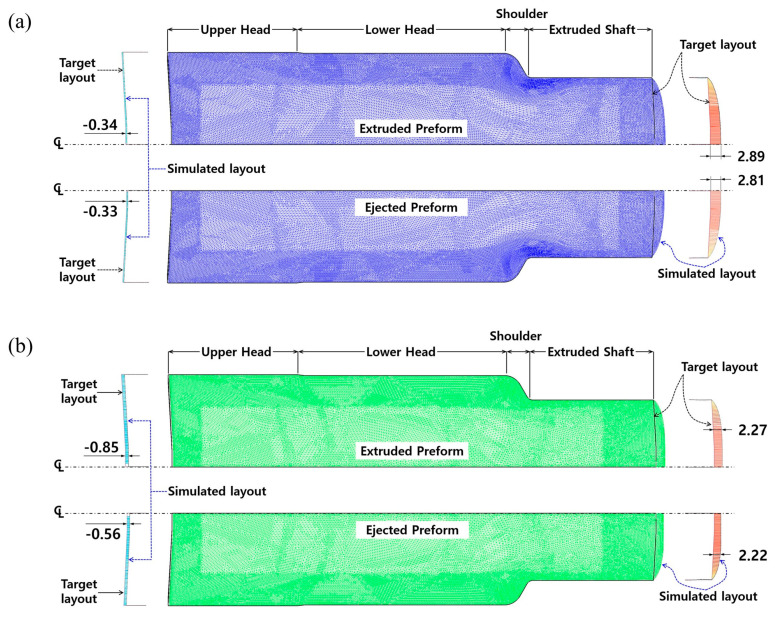
Dimension variation (unit: mm): (**a**) target layout vs. plastic FE simulation, and (**b**) target layout vs. elasto-plastic FE simulation.

**Figure 16:**
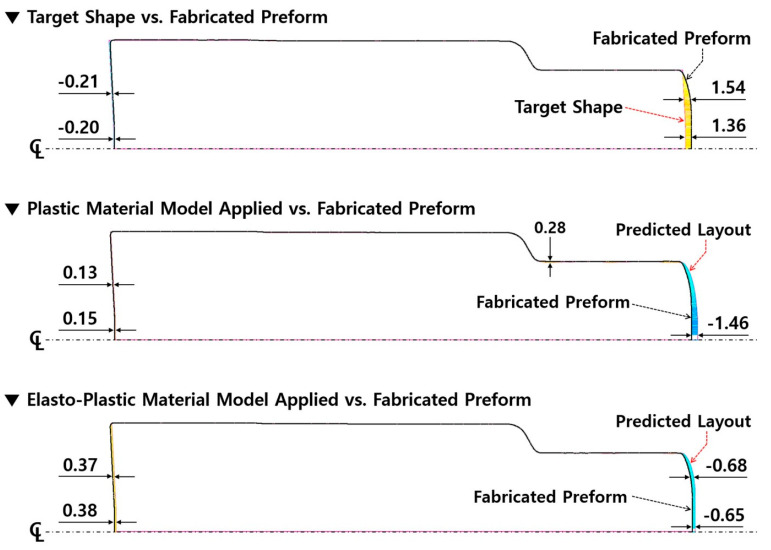
Comparison of dimensional suitability between fabricated and numerically simulated preforms (unit: mm).

The three-dimensional numerical simulations related to the forward extrusion process and the ejection operation were continuously carried out, during which the plastic and the elasto-plastic material models were considered for describing the deformation behavior of the heat-treated and the phosphophyllite-coated initial billet, and the obtained results indicated that the whole shapes were well forged. However, it was necessary to identify the dimensional variations caused by the difference in the material models adopted to the numerical simulations. For the case in which the plastic material model was adopted, based on the required dimensions shown in Figure 1b and the deformed preform shown in Figure 8, the fully extruded workpiece had dimensional errors of −0.34 mm at the top end of the upper head area, and 2.89 mm at the extruded shaft end before ejection. Upon completion of the ejection process, the errors of −0.33 mm at the top end and 2.81 mm at the shaft end were predicted, as shown in Figure 15a. The round-type excessive protrusion around the shaft end was also observed, and it was attributed to the frictional behavior between the workpiece and the middle (extrusion) die. That is, when the plastic material model was applied to the numerical simulations with regard to the forward extrusion and the ejection, the post deformation induced by the counter punch stroking was found to occur so rarely as to be negligible. In the case that the elasto-plastic material model was assumed as the deformation behavior of the workpiece material, the deformed preform had the dimensional differences of −0.85 mm at the top end of the upper head area, and 2.27 mm at the extruded shaft end after the forward extrusion, as shown in Figure 15b. However, it was revealed that the variations were slightly improved to be −0.56 mm at the top end and be 2.22 mm at the shaft end. In particular, the excessively compressed workpiece at the top end was slightly recovered by the elasto-plastic deformation behavior during the ejection operation. Further, the elliptic-type excessive protrusion appeared around the extruded shaft end, so it was noted that the post deformation occurred during the ejection from the counter punch operation. However, as indicated in Figure 15, the dimensional errors based on the target geometry were predicted to be under approximately 1.00 mm at the top section of the upper head, and less than 2.81 mm at the shaft end of the ejected preform, and the deformed layouts at the shaft end were so dissimilar as to excessively protrude with round-type and the elliptic-type profiles. Accordingly, in order to compare the differences in view of the dimension and the deformed feature between the numerically simulated and fabricated preforms, first, the fabricated preform was fully scanned using an optical 3D scanner (Rexcan IV of Solutionix, Seoul, Republic of Korea). Then, the captured image was used in a series of comparative investigations conducted by adopting an image processing software (Geomagic Qualify of 3D Systems), as shown in Figure 16. Moreover, because the preform used in the three-dimensional optical scanning had already been pulled out from the die cavity, the comparative objects were taken as the fully ejected workpieces in the numerical simulations. 

When the fabricated preform was set as the reference geometry and the shoulder profiles were aligned with each other using the comparative object, the maximal dimensional errors with the required target shape illustrated in Figure 1b were evaluated to be −0.21 mm at the top area of the upper head and 1.54 mm at the shaft end of the extruded preform. Those with the deformed preform were valued to be 0.15 mm at the top area and −1.48 mm at the shaft end by applying the plastic material model, and those with the predicted shape were estimated to be 0.38 mm at the top area and −0.68 mm at the shaft end by adopting the elasto-plastic material model. These quantitatively compared results are summarized in Table 5. In particular, it was observed that the preform actualized through the cold forward extrusion experiment also protruded excessively for the same reasons analyzed when using the numerical simulations, but it was revealed that the deformed layout around the extruded shaft end was very similar to the numerically predicted one achieved by applying the elasto-plastic material model. Considered comprehensively, in spite of the fact that the numerically simulated preform and the actually fabricated one slightly differed from the required dimensions and feature of the target object, it was demonstrated that the FEM-based numerical simulations achieved by applying the elasto-plastic material model can be more suitable for visualization in order to be well matched with the cold forged preform.

### 4.3. Hardness and Residual Stress

On the other hand, the hardness was measured to investigate the influence of the spheroidizing and annealing heat treatment on the AISI 1035 workpiece material, and to evaluate the change created by the cold forward extrusion. The test equipment used for this was the Vickers micro-hardness measurement apparatus (AMT-X7FS, automatic Vickers hardness test system of Matsuzawa, Nagano, Japan), and a loading condition of 25g_f_ was applied. The Vickers micro-hardness was measured at the nine pre-defined points on the longitudinally half-cut surface of the raw specimen, and the average value was about HV_25_ 174.8, with a range of HV_25_ 168.2~188.1. Further, at the same points of the heat-treated specimen, the average value was approximately HV_25_ 136.8 with a range of HV_25_ 134.6~142.8. Accordingly, it was ensured that the hardness decreased by nearly HV_25_ 38.0 with the spheroidizing and annealing heat treatment, and its distribution was more uniform without a large deviation. The measured Vickers micro-hardness results are summarized in Table 6. 

**Figure 17:**
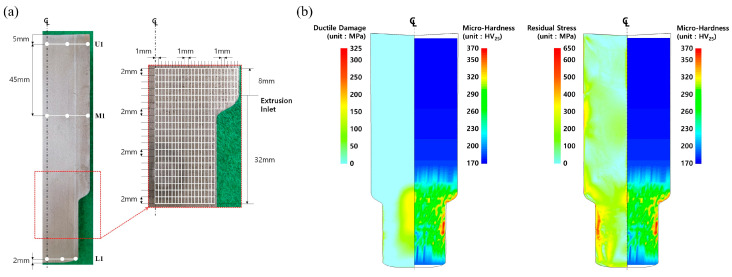
Vickers micro-hardness distribution of preform fabricated by cold forward extrusion: (**a**) detailed grid structures for measuring micro-hardness; (**b**) comparison of Vickers micro-hardness distribution between ductile damage and residual stress.

Moreover, the Vickers micro-hardness on the longitudinally half-cut specimen of the manufactured preform was also measured at each point on the grid map as depicted in Figure 17a. Three points were selected, one 5 mm away (U1) from the top area of the upper head, one 45 mm away (M1) from these points, and one 2 mm away (L1) from the bottom end of the extruded shaft, then the micro-hardness was measured to have the average values of about HV_25_ 172.9, HV_25_ 176.8, and HV_25_ 193.8, respectively. In addition, to investigate the Vickers micro-hardness at the extruded part in which the plastic deformation by the cold forward extrusion operation actually occurred, the tight grid structure with even intervals of 1.0 mm along the radial direction and 2.0 mm along the longitudinal one is illustrated in Figure 17a, and the measured result is presented in Figure 17b. Altogether, it was denoted that the measured Vickers micro-hardness is more well matched with the residual stress distribution than the predicted plastic deformation damage. 

### 4.4. Microstructure and Residual Stress

Because the AISI 1035 raw workpiece was heat-treated through spheroidizing and annealing, it could be anticipated that the residual stress inherent in the raw workpiece material was influenced. In order to identify and compare the difference of the residual stress in view of the dislocation density (or the internally stored strain energy) as the influence of the heat treatment on the workpiece material, the IQ (image quality) map and the IPF (Inverse Pole Figure) map, as well as the KAM (kernel average misorientation) map, were examined. To this end, the EBSD (electron backscatter diffraction) analysis was carried out by using the FE-SEM (field-emission scanning electron microscopy) of TESCAN MIRA3 at an acceleration voltage of 15 kV, after which the obtained EBSD data were analyzed by adopting the orientation imaging microscopy software (TSL OIM 7.3.1) [39]. 

**Figure 18:**
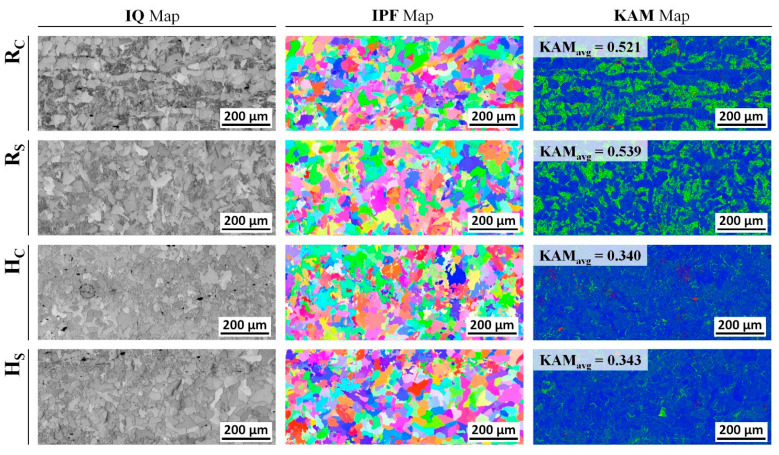
EBSD IQ map and IPF map, and KAM map, of raw and spheroidized-annealed specimen.

Figure 18 shows the IQ map and IPF map, as well as the KAM map, with sections denoted as R_C_ and R_S_ as well as H_C_ and H_S_ in Table 6. Regarding the influence of the heat treatment, no dramatic change in the dislocation density could be clearly observed from the EBSD IQ map, and the grain boundary was well identified from the IPF map. However, around the central core (R_C_) section and the outer surface (R_S_) region of the raw workpiece, the typical medium carbon steel characteristics were observed, and the average KAM values were calculated to be 0.52 and 0.54, respectively. For the influence of the spheroidizing and annealing heat treatment, the average KAM values were slightly decreased to be 0.33 at the part of H_C_ and 0.34 at the area of H_S_. Therefore, it was ensured that the average KAM value for the heat-treated specimen was decreased by roughly 40% in the case of comparing the value to that of the raw workpiece. Due to the fact that the KAM value is proportional to the dislocation density or the internally stored strain energy, and the fact that the value was decreased by nearly 40%, the ductility of the spheroidized and annealed AISI 1035 billet material was found to be increased.

**Figure 19:**
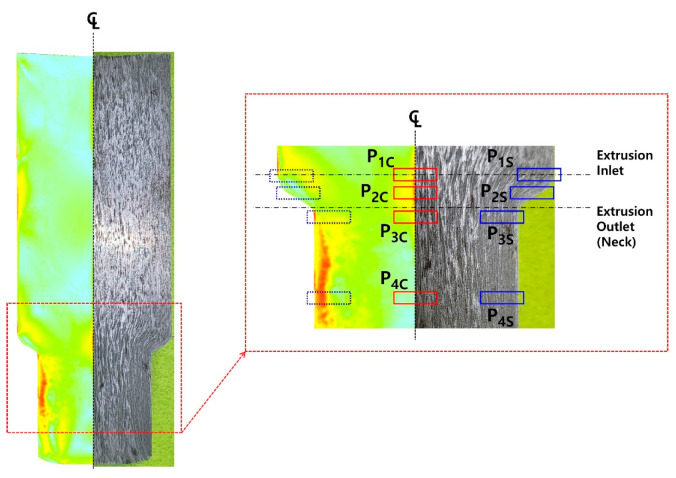
Measuring sections for EBSD analysis of cold forward-extruded preform.

**Figure 20:**
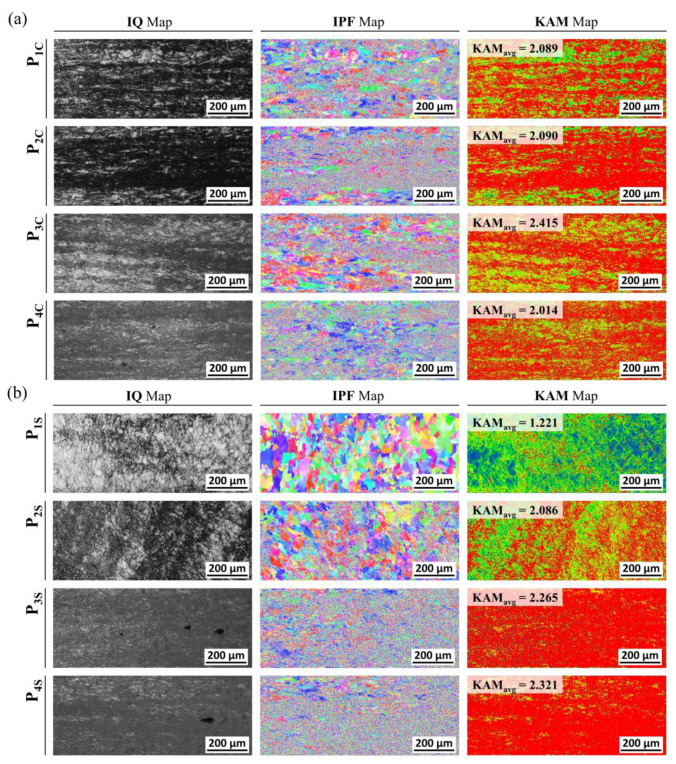
EBSD IQ map and IPF map, and KAM map: (**a**) on central core regions; (**b**) on near outer surface regions.

For the cold forward-extruded preform, with consideration of the residual stress level and the plastic deformation damage degree predicted through the numerical simulations in which the elasto-plastic material model was adopted, the IQ map and the IPF map, along with the KAM map, were observed at each region denoted in Figure 19. Regarding the EBSD IQ maps and IPF maps on the central core region as illustrated in Figure 20a, it can be seen that the dislocation density at each section (P_1C_, P_2C_, P_3C_, P_4C_) was distinctly increased through a comparison with that at H_C_ of the heat-treated initial workpiece, and that the grain boundary was not clearly distinguished. With regard to the KAM map, the values of KAM_avg_ were calculated to be 2.09 at P_1C_, 2.18 at P_2C_, 2.42 at P_3C_, and 2.51 at P_4C_, while those from P_1C_ to P_4C_ were highly increased in comparison to the value of about 0.34 at H_C_ of the heat-treated initial workpiece. Furthermore, the EBSD analysis was also conducted around the outer surface regions as depicted in Figure 20b. Around the outer surface part within the cold forward-extruded preform, the IQ maps from P_1S_ to P_4S_ explained that the dislocation density was evidently increased as the cold forward extrusion progressed, and the IPF maps indicated that the grain boundaries were clearly distinguished at P_1S_ to P_2S_, but these boundaries at P_3S_ to P_4S_ were difficult to clarify after passing the extrusion outlet (that is, the neck section). In view of the KAM map, the value of KAM_avg_ at P_1S_ was observed to be 1.221, and therefore larger than that of 0.343 at H_C_ of the spheroidized and annealed specimen, and it was revealed that the value is highly distributed with 2.086 at P_2S_, 2.265 at P_3S_, and 2.321 at P_4S_. 

With respect to the EBSD IQ map, it was ensured that the dislocation density at the central core sections and the outer surface regions was largely increased by comparing with the density of the heat-treated specimen. In view of the IPF map for the deformed preform after passing the extrusion outlet, the discrimination of the grain boundary at P_3S_ and P_4S_ around the outer surface parts was harder than that at P_3C_ and P_4C_ around the central core regions, and the grains were considered to be more severely distorted and densely arranged than those at other sites. Moreover, it was pointed out that the KAM values reached roughly 2.063 as the whole average of the observed KAM_avg_, which was increased by about 516% when compared to 0.335 for the heat-treated specimen. Considering the results extracted from the EBSD analysis, the plastic deformation around the central core region was regarded to be mainly derived and accumulated by the compressive loads induced from the reaction forces on the circumferential-sloped extrusion die, and the permanent shape change around the outer surface section was considered to have critically occurred because of the compressive and shear deformation due to the reduction in the cross-sectional area.

## 5. Conclusions

This study investigated the residual stress and microstructure, as well as the Vickers micro-hardness and the plastic deformation damage, within a preform fabricated by the cold forward extrusion process. The AISI 1035 cold-drawn medium carbon steel material was selected to produce the preform, and the influence of the spheroidizing and annealing heat treatment on the mechanical properties and the material characteristics were evaluated. Using Voce’s constitutive equation based on the uniaxial compression test results, a series of FEM-based numerical simulations wherein the deformation behavior was assumed to use either the plastic or the elasto-plastics material models, were conducted. Furthermore, the preform produced through the cold forward extrusion was fully captured, and the scanned image was compared with the geometry of the numerically simulated preform. The Vickers micro-hardness was measured and compared to the plastic deformation damage and the residual stress which were predicted using the three-dimensional numerical simulations. Additionally, the EBSD analysis was carried out in order to investigate the dislocation density and the microstructural characteristics. The results of this study can be summarized as follows:

1. Regarding the influence of the spheroidizing and annealing on the mechanical properties and the material characteristics of the AISI 1035 cold-drawn medium carbon steel, the two-phase microstructure of the ferrite and the pearlite with the lamellar structure of the ferrite and the cementite (Fe_3_C) were observed to be transformed such that the polygonal ferrite was freely distributed, and the decomposed pearlite was partly broken and partially spheroidized. For the heat-treated material, the micro-hardness was estimated as roughly HV_25_ 136.8, and the compressive strength was measured to be 638.30 MPa.
2. The preform manufactured through cold forward extrusion was fully scanned, and the captured image was compared with the numerically obtained configurations in the case where the plastic material and the elasto-plastic material models were adopted. The preform was then virtualized by adopting the elasto-plastic material model matched with the fabricated product well. Further, through the numerical simulations adopted with the elasto-plastic material model, the residual stress inherent within the preform was predicted to be nearly 120 MPa on the whole preform.
3. The micro-hardness was observed to be high around the region in which the extrusion actually progressed, and the predicted residual stress in terms of the distribution tendency was better expressed with the measured micro-hardness than the calculated plastic deformation damage.
4. Based on the IQ map and the IPF map extracted through the EBSD analysis, it was found to be extremely difficult to discriminate the grain boundary around the cold forged region which was due to the high-degree of dislocation density. With regard to the average KAM value, it was denoted that the KAM_avg_ was increased by about 516% compared to the heat-treated initial billet.
5. Because severely accumulated dislocation density and extreme KAM_avg_ were observed, it was regarded that the considerable residual stress within the preform realized in this study was actually inherent. Therefore, it was considered that an additional heat treatment, with which the dislocation density and the residual stress can be mitigated, is necessary to use the preform as the intermediate product for manufacturing the drive shaft used for the industrial hydraulic pump.

## Figures and Tables

**Table 1:** Chemical composition of AISI 1035 medium carbon steel material as received [unit: wt%].

C	Mn	Si	P	S	Fe
0.350	0.800	0.275	0.003	0.005	Bal.

**Table 2:** Grain size of raw and heat-treated AISI 1035 specimens.

	Min.	Max.	Average
Raw Material	7.27 µm	11.42 µm	8.84 µm
Spheroidized-Annealed Material	6.67 µm	12.31 µm	9.36 µm

**Table 3:** Mechanical properties obtained by uniaxial compressive tests of AISI 1035 specimens.

Properties	Raw Material		Heat-Treated Material	
	Engineering	True	Engineering	True
Young’s Modulus (GPa)	196	196	196	196
Poisson’s Ratio	0.29	0.29	0.29	0.29
Yield Strength (MPa)	362.32	342.53	299.20	289.65
Ultimate Strength (MPa)		723.99		638.30

**Table 4:** Prediction and comparison on true stress-strain curve using various flow stress models.

Flow Stress Model	Formulation		Fitted Equation	
Hollomon	$\bar{\sigma }=K_{H}{\bar{\varepsilon }}^{\;n_{H}}$	(MPa)	$\;\;\bar{\sigma }=689.1\;{\bar{\varepsilon }}^{\;0.165}$	(MPa)
Swift	$\;\;\bar{\sigma }=K_{S}{\left({\bar{\varepsilon }}_{0}+\bar{\varepsilon }\;\right)}^{n_{S}}$	(MPa)	$\;\;\bar{\sigma }=690.4{\left(0.00393+\bar{\varepsilon }\;\right)}^{0.171}$	(MPa)
Ludwik	$\;\;\bar{\sigma }={\bar{\sigma }}_{0}+K_{L}{\bar{\varepsilon }}^{\;n_{L}}$	(MPa)	$\;\;\bar{\sigma }=-758.4+1436.7\;{\bar{\varepsilon }}^{\;0.065}$	(MPa)
Voce	$\;\;\bar{\sigma }=K_{V1}+K_{V2}e^{-n_{V}\bar{\varepsilon }\;}$	(MPa)	$\;\;\bar{\sigma }=633.9-338.5\;e^{-7.937\;\bar{\varepsilon }\;}$	(MPa)
[Note]	$K_{H},\;K_{S},K_{L},K_{V1},K_{V2}$		: the material constants	
	${\bar{\sigma }}_{0},\;{\bar{\varepsilon }}_{0}$		: the initial stress and strain	
	$n_{H},\;n_{S},n_{L},n_{V}\;$		: the work-hardening coefficients	

**Table 5:** Summary of dimensional compatibility of preform obtained through forward extrusion and ejection operations (unit: mm).

Properties	Target	Plastic		Elasto-Plastic		Experiment
		Extrusion	Ejection	Extrusion	Ejection	
Whole Length	(134.15)	136.73	136.65	135.53	135.62	135.38
Upper Head Diameter	Ø51.0	Ø50.99	Ø50.99	Ø50.99	Ø51.05	Ø51.04
Lower Head Diameter	Ø50.6	Ø50.59	Ø50.59	Ø50.59	Ø50.65	Ø50.64
Shaft Diameter	Ø37.0	Ø36.67	Ø36.71	Ø36.80	Ø36.83	Ø36.58
Extruded Shaft Length	34.5 ± 1.0	34.52	34.52	34.42	34.42	34.35

**Table 6:** Measured Vickers micro-hardness before and after spheroidizing-annealing of AISI 1035 workpiece material (unit: HV_25_).

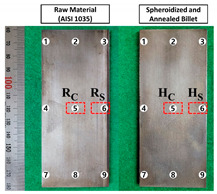	**Measured Point**	**Raw**	**Heat-Treated**
	1	178.2	136.2
	2	170.3	137.8
	3	171.1	137.7
	4	188.1	135.0
	5	168.2	134.6
	6	172.8	138.2
	7	178.1	139.9
	8	172.1	136.0
	9	174.4	142.8
	Average	174.8	136.8

## Data Availability

New data were not created or analyzed in this study. Data sharing is not available for this article.
